# Collectanea Medica: Consisting of Anecdotes, Facts, Extracts, Illustrations, &c.

**Published:** 1823-04

**Authors:** 


### COLLECTANEA MEDICA:

CONSISTING OF

### ANECDOTES, FACTS, EXTRACTS, ILLUSTRATIONS, &c.

*Relating to the History or the Art of Medicine, and the Collateral Sciences.*

---

Floriferis, ut apes, in saltibus omnia libant,
Omnia nos, itidem, depascimur aurea dicta.

---

ART. I.—*Antidotes.* [From Dr. PARIS's *Pharmocologia*, 5th Edit.]

POISONS differ materially from each other, not only with respect to the modes in which they produce their effects in relation to the several vital organs, but with respect to their application. Some of those, for instance, which are speedily fatal if introduced into a wound, may be taken into the stomach with complete impunity,—as in the instance of the venom of the viper and other snakes, which appears to exert no influence on the stomach: others, on the contrary, display their deleterious action on the stomach alone,—such as caustic acids and alkalies, corrosive sublimate, and other chemical poisons: while others, again, are equally destructive, whether applied to the inner surface of the stomach, or to the lower intestines, in the form of clyster, or even to the mucous membrane of the mouth or nose; to the eye; to the vagina and orifice of the uterus, or to an abraded portion of the skin. There is, moreover, a class of substances which may be termed *aërial* poisons; for they may exist in the state of gas, or be held dissolved in the atmosphere, and be received by respiration, or by the mucous membranes of the nose and throat; the saliva may also thus become the medium for transferring various subtile poisons from the atmosphere to the animal body. This is well illustrated by the fact of the transfer of metallic influence, as related in the case of a gentleman in perfect health, who became salivated in consequence of sitting for one hour by the side of a person who was in a state of mercurial ptyalism, in order to give him a lesson in botany.

It also deserves notice, that a poison acts with different degrees of force and celerity in different parts of the same tissue: its absorption, for instance, would appear to be energetic in proportion to the number of veins, although several apparent exceptions to this law might be adduced; and it is evident that the plethoric state of the part, with respect to its blood-vessels, has a considerable share in modifying the effects. This observation, however, has no relation to those poisons which operate on the system through the sympathetic communication of the nerves. Mr. Brodie, for instance, found that the poison of bitter almonds acted more speedily when applied to the tongue than when injected into the intestine, though the latter presents a much better absorbing surface.

Foderé, in the fourth volume of his Médecine Legale, arranges poisons according to their action on the living system, and which, with a slight alteration in the order of the classes, has been adopted by Orfila, and most other writers on Toxicology. Poisons are thus reduced into six classes: viz.—1. *Corrosive or Escharotic*, as the preparations of Mercury, Arsenic, Antimony, Copper, Tin, Zinc, Silver, Gold, and Bismuth; the concentrated Acids, and caustic Alkalies and Earths; Cantharides; Glass and Enamel Powder. 2. *Astringent Poisons*, of which the preparations of Lead constitute the only species. 3. *Acrid or Rubefacient Poisons*, which, with a few exceptions, are furnished by the vegetable kingdom, as certain drastic Purgatives, Hellebore, Euphorbium, &c. 4. *Narcotic Poisons*,—Opium, Henbane, the Cherry-laurel, Stramonium, &c. 5. *Narcotico-Acrid*, embracing such articles as produce the united effects of the two former, and which constitute some of the most deadly poisons, as the Ticunas, Nux-vomica, Belladonna, Tobacco, Hemlock, Digitalis, &c. 6. *Septic Poisons*,—Contagious Miasmata, Putrid Exhalations from Animal Matter, Sulphuretted Hydrogen, the Venom of the Viper, &c. If we are to found our classification upon views strictly pathological, the above arrangement is perhaps as eligible as any that could be proposed, and it has certainly the obvious advantage of bringing together those individual substances which occasion effects somewhat analogous upon the living system; and yet such a plan will not enlighten our therapeutical doctrines. Arsenic and corrosive sublimate are, for instance, both corrosive poisons, but they differ from each other materially in their physiological action; and, when swallowed, require, for the preservation of the individual, a very different system of treatment. It is from the views of their physiological and chemical action that our curative indications are to be chiefly derived; and the able and interesting researches of Mr. Brodie enable us, in a very satisfactory manner, to construct an arrangement by which the treatment of poisons may be at once rendered more efficacious and simple: thus poisons may be said to act—

I. Through the medium of the Nerves, without being absorbed.

*a.* By destroying the functions of the brain.—Alcohol. Essential Oil of Almonds. Juice of Aconite. Oil of Tobacco. Opium?

*b.* By rendering the heart insensible to the stimulus of its blood.—Infusion of Tobacco.

II. By entering the Circulation, and acting through that medium, with different degrees of force, on the Heart, Brain, and Alimentary Canal.

Arsenic. Emetic Tartar. Muriate of Baryta. The Woorara, and all poisons which act by external application, as the venom of the viper, &c.

III. By a local action on the Mucous Membrane of the Stomach.

Corrosive Sublimate of Mercury. Acids and Alkalies. Mechanical Irritants, as diamond dust? powdered glass?

It had been long admitted that a poison may produce death by acting on the extremities of the nerves of the stomach and intestines, without being absorbed; but to the experimental labours of Mr. Brodie we are indebted for our present correct views upon the subject. It was observed by Bichat, (and the observation has been confirmed by Brodie,) that the influence of the brain is not directly necessary to the action of the heart; for that, when the functions of the brain are destroyed,—even when the head is removed,—the heart continues to contract for some time afterwards, and then ceases only in consequence of the suspension of respiration, which is under the direct influence of the brain. Assuming this as a principle, it will easily appear that certain poisons may, by affecting the brain, paralyze the muscles of respiration, and occasion death by suffocation. Mr. Brodie accordingly found that, by the administration of a large dose of alcohol to a rabbit, the pupil of its eyes became dilated, the extremities convulsed, and the respiration laborious; and that this latter function was gradually performed at longer and longer intervals, and that it at length entirely ceased. Two minutes after the apparent death of the animal, he opened the thorax, and found the heart acting with moderate force and frequency, *circulating dark-coloured blood.* He then introduced a tube into the trachea, and produced artificial respiration by inflating the lungs; and found that, by these means, the action of the heart might be kept up to the natural standard, as in an animal from whom the head is removed. The same phenomena resulted from the injection of two drops of the essential oil of bitter almonds, diffused in half an ounce of water, into the rectum of a cat; and from the application of the empyreumatic oil of tobacco to the tongue and rectum of cats and dogs. Now, it is obvious that the functions of the brain are immediately disordered by the influence of these poisons on the tongue, stomach, and lower bowels of animals, so instantaneously, that it is impossible that absorption should have already taken place. The poisons above enumerated appear to affect the brain only, and through it the organs of respiration, leaving the powers of the circulation unimpaired: there are, on the contrary, certain substances which act on the heart at once; such is the infusion of tobacco, which suspends its action even before the animal ceases to respire, and kills by producing syncope. The same effects are occasioned by the two species of upas from Java, called *upas antiar* and *upas tieuté.* In these cases, it is probable that the spinal marrow is primarily affected, which recent experiments have shown to have an intimate relation with the action of the heart. We next come to speak of those poisons which enter the circulation, and act through that medium on the heart, brain, and alimentary canal. These organs, however, are affected in very different degrees by different poisons. Mr. Brodie has shown that vegetable poisons, although when introduced into the alimentary canal affect life, in consequence of the nervous sympathy which subsists between these surfaces and the common sensorium, yet, that the same poisons applied externally to a wound produce their effects exclusively through the medium of the circulation, being conveyed to the brain only by mixing with the blood in the vessels, and not by being conveyed through the absorbents; for a ligature upon the great vessels prevents them from producing their deleterious effects: whereas a ligature upon the thoracic duct, or general canal, through which all the absorbents pour their contents into the blood, does not in the least retard or prevent the operation of the poison. There are also several of the mineral poisons, which, whether introduced into the stomach or applied externally to a wound, poison the animal, in consequence of being carried into the circulation. It had long been supposed that arsenic occasioned death by inflaming the stomach; but Mr. Brodie has clearly demonstrated that its influence arises from its being absorbed, and that it must be regarded as a vital rather than as a chemical agent. In the first place, he has found the inflammation of the stomach in several cases so slight, that on a superficial examination, it might have been easily overlooked; and in most of his experiments with arsenic, death took place in too short a time to be considered as the result of inflammation; and, in the next place, in whatever manner the poison is applied, whether externally to a wound, or internally to the membrane of the stomach, the inflammation is confined to the stomach and intestines: and, indeed, it is commonly more violent, and even more immediate, when applied to a wound, than when internally administered; and it also precedes any inflammation of the wound. This important fact was proved by an experiment made by Mr. Hunter and Sir Everard Home, and subsequently by the repeated investigations of Mr. Brodie. It deserves notice, however, that, although the affection of the stomach and intestines from this poison is not, under ordinary circumstances, the cause of death, it may be so in some instances, where the animal survives the effects produced on the organs which are more immediately subservient to life. Mr. Henry Earle communicated to Mr. Brodie an interesting case of this description, of a woman in St. Bartholomew's Hospital, who had recovered from the immediate symptoms occasioned by the ingestion of arsenic, but died at the end of four or five days; when, upon dissection, there appeared extensive ulcerations of the stomach and bowels.

Baryta, and still more actively its muriate, produce similarly deleterious effects, whether internally or externally applied; and, like arsenic, it affects life by rendering the heart insensible to its blood. The same observations will apply to the operation of tartarized antimony.

Corrosive sublimate of mercury, and some other poisons, produce their destructive effects, not by impression on the nervous system, nor by entering the circulation, but by inducing local injury on the mucous membrane of the stomach.

Having thus offered a summary of our present views respecting the physiological action of poisons, we are prepared to lay down a general plan of treatment, which, it will be seen, can only be successful when conducted on principles strictly conformable with the just notions which the preceding experiments have so satisfactorily established.

Where a poisonous substance has, either through accident or design, found its way into the alimentary canal, three important indications are, if possible, to be fulfilled: and under these heads I shall offer such observations as may serve to instruct the practitioner in the philosophy of the general treatment, reserving the details to be observed in that of each poison, for more particular notice in the second part of the work, where the history of these substances will be individually considered. The indications to which I allude are the following: viz.—

1. *The immediate ejectment of the poison from the body, by the operation of vomiting and purging.*—Whatever may be the nature of the poison, we should endeavour, with all possible expedition, to eject it from the body; and upon the promptness with which this is effected the safety of the patient will generally depend; for the dangerous effects of such substances advance in a very increasing ratio, with the time they remain in contact with a living surface. A question may arise, whether in some cases it would not be judicious to attempt, in the first instance, the neutralization or decomposition of the poison. Where a mineral acid or a caustic alkali has been swallowed, it would undoubtedly be right to neutralize and dilute it as soon as possible, and then to excite vomiting, which may be advantageously effected by thrusting the finger down the throat, or by tickling the internal fauces with a feather. Where an emetic is at hand, whatever may be its nature, it should be promptly given; but, if circumstances will allow us the opportunity of selection, antimonials, ipecacuanha, &c. should be rejected, and sulphate of zinc and sulphate of copper, for several reasons, be preferred: in the first place, they do not require much dilution for their action,—a circumstance of no small importance in the treatment of poisons that act by being absorbed; in the next place, they are extremely expeditious,—a dose of fifteen or twenty grains producing almost instantaneous vomiting, without exciting that previous stage of nausea which so frequently characterizes other emetics, and which occasions a state of the vascular system highly favourable to the function of absorption. After all has been ejected which the operation of vomiting can effect, we are to proceed to the fulfilment of the second indication, viz.—

2. *The decomposition of the remaining portion, and the adoption of measures best calculated to obviate its absorption.*—Where the substance is in a solid form, and acts by absorption, we should be very cautious how we favour its solution; while, if it exists in a liquid state, our object must be to render its active portion insoluble: this problem involves a series of questions which are wholly chemical. In order to prevent, or retard, the absorption of the active matter, we must, to a great degree, depend upon the agency of vital adjuvants. This latter indication, however, does not apply to corrosive sublimate and other substances which act upon the stomach locally, and are not absorbed. Copious dilution also, in such cases, will frequently disarm the poison of its virulence; but it should be followed as quickly as possible by vomiting. In cases where the poison requires to be absorbed before it can display its energies, it would be generally unsafe to administer any solvent. Alkaline solutions and magnesia, in cases of the ingestion of arsenic, accelerate its fatal effects, by promoting its solution; whereas lime, or its carbonate, has an opposite tendency, in consequence of the insolubility of arsenite of lime: so again, Orfila has shown that the pernicious qualities of the muriate of baryta are counteracted by the administration of any soluble sulphate, which renders the former substance insoluble. In cases where verdigris has been swallowed, the administration of vinegar greatly increases its virulence, as M. Drouard has ascertained, by converting the substance into a soluble acetate of copper. This view of the subject will explain why the pure earth of baryta is so slow, and comparatively inert, in its effects upon the system, while its muriate is distinguished by the extreme rapidity and virulence with which it operates. The propriety of administering vinegar, lemonade, and different acid potations, in order to counteract the baneful effects of opium, which has been so often questioned, will thus also receive ample explanation. It must appear that, if any quantity of the substance of opium remain in the primæ viæ, acid or mucilaginous drinks will, by favouring its solution and absorption, accelerate its fatal effects; but, should it have been previously ejected from the stomach, that then the anti-narcotic influence of a vegetable acid may remove the consecutive stupor and delirium. * * * *

Chardin, in his Travels through Persia, informs us that, when a Persian finds himself in a distressed situation, he has recourse to a piece of opium as large as the thumb, and that immediately afterwards he drinks a glassful of vinegar; by which he is thrown into a fit of laughter, terminating in convulsions and death.

With regard to the use of antidotes, it has been already stated how little they are to be depended upon: in certain cases, however, we are bound to acknowledge their power, but they should be very rarely trusted, unless subsequent to, or in conjunction with, the operation of an emetic. In many cases, the effects of this latter remedy may be promoted by the ingestion of liquids holding the particular antidote in solution,—a practice which offers the double advantage of accelerating the elimination of the poison, and at the same time of decomposing any which may remain. Orfila has fully established the fact of albumen being a counterpoison to corrosive sublimate: vomiting may, therefore, be very judiciously promoted, in cases of such poisoning, by water holding the white of egg in solution. With equal effect, where verdigris has been swallowed, sugared water may be used as a diluent to encourage emesis; and muriate of soda in solution will be found the most efficient antidote to nitrate of silver; and sulphate of magnesia to acetate of lead. Where an emetic salt, like tartarized antimony, has been taken, copious dilution with common water will, in general, so provoke vomiting, as to render it its own antidote; but it may be useful to remember that the infusion of galls, and, according to Berthollet, the decoction of bark, at the temperature of from 30° to 40° Fahr., have the power of decomposing it; while Orfila considers milk the most efficient counterpoison to the sulphate of zinc.

Having ejected from the stomach all the poisonous matter we can by vomiting, and attempted to decompose what remains, we are to pursue such measures as may be calculated to prevent the absorption of the poison into the circulation: it has been already observed that, on this account, nauseating emetics should be avoided. Venesection proves one of the most powerful means of exciting the function of absorption; hence, in poisoning by arsenic, such an expedient should never be recommended while a particle of that substance remains in the body: where corrosive sublimate has been swallowed, the same precaution is unnecessary. The last indication which remains to be fulfilled is—

3. *To anticipate the occurrence of the consecutive phenomena, and to combat them by an appropriate treatment.*—This is to be conducted on the general principles of therapeutics: the treatment must necessarily vary in each particular case. When the exhaustion of nervous energy is to be feared, (as after poisoning by prussic acid,) ammonia and other diffusible stimulants will furnish the best resource: where, on the other hand, inflammatory action is to be anticipated, it is unnecessary to detail the plan of treatment which may be adopted with the greatest chance of success. In cases where the nervous system is stupified, the symptoms may be combated by vegetable acids, infusion of coffee, &c.; but where it is in a state of preternatural excitement, recourse must be had to opiates.

